# Nanotube Size Alters Corrosion Behavior, Mechanical Properties, and Biological Interactions of Anodized Ti6Al7Nb

**DOI:** 10.1021/acsomega.5c07922

**Published:** 2025-12-29

**Authors:** Yasar Kemal Erdogan, Merve Izmir, Olgu Cagan Ozonuk, Cem Bayram, Batur Ercan

**Affiliations:** † Department of Biomedical Engineering, Isparta University of Applied Science, 32200 Isparta, Turkey; ‡ Biomedical Engineering Program, 52984 Middle East Technical University, 06800 Ankara, Turkey; § Department of Metallurgical and Materials Engineering, Middle East Technical University, 06800 Ankara, Turkey; ∥ School of Materials Science and Engineering, 54761 Nanyang Technological University, 639798, Singapore; ⊥ Institute for Graduate Studies in Science and Engineering, Nanotechnology and Nanomedicine Division, 37515 Hacettepe University, 06800 Ankara, Turkey; # BIOMATEN, METU Center of Excellence in Biomaterials and Tissue Engineering, 06800 Ankara, Turkey

## Abstract

The fabrication of oxide-based nanotubes on implant surfaces has garnered significant attention in orthopedics due to their enhanced surface area and nanostructured roughness, which promote integration with bone tissue. However, studies investigating the effects of varying pore diameters while maintaining constant oxide layer thickness remain limited, despite the potential of pore size to influence mechanical performance, cellular responses, and the long-term success of implants. In this study, we fabricated nanotube structures with fixed lengths—short (∼1 μm, S) and long (∼4 μm, L)—but with varying pore sizes, to gain a deeper understanding of how nanotube dimensions and nanostructured topography influence corrosion behavior, mechanical properties, and *in vitro* cellular interactions. Scanning electron microscopy (SEM) and atomic force microscopy (AFM) analyses confirmed that a uniform nanostructure with a roughened surface was successfully achieved through anodization. Corrosion experiments revealed that the short nanotubes formed at 10 V (NT10-S) exhibited reduced corrosion, approximately 40% and 49% lower than that of the long nanotubes formed at 10 V (NT10-L) and nonanodized (NA) control, respectively. Moreover, the NT10-S samples exhibited the highest critical load, measured as 0.16 ± 0.02 N in the microscratch test. Notably, osteoblast proliferation was enhanced by 47% on NT10-S samples after 5 days of culture compared to NA surfaces. This study provides valuable insights into how nanotube length, pore diameter, and surface nanostructured topography anodized surfaces influence both chemical and biological responses, offering critical guidance for the design of next-generation orthopedic implants.

## Graphical abstract

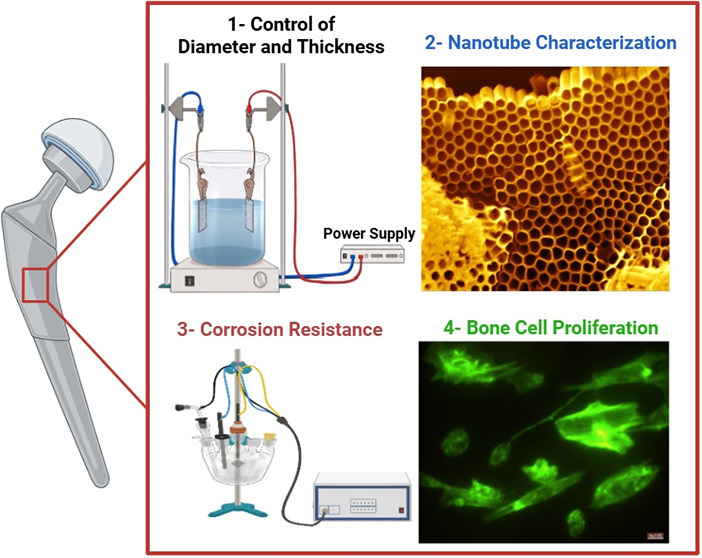

## Introduction

Ti6Al7Nb is among the latest-generation implant materials, particularly in orthopedic and dental applications, due to its biocompatibility and favorable mechanical properties.^1^ This alloy exhibits high strength and a lower elastic modulus compared to other metallic materials commonly used in orthopedics. A reduced elastic modulus decreases the risk of stress shielding, a phenomenon where the implant bears a significant portion of the load, leading to bone resorption.^2^ Additionally, unlike the widely used Ti6Al4 V alloy, Ti6Al7Nb replaces vanadium with niobium, which is considered safer for long-term implantation in the body.^3^ Ti6Al7Nb is well tolerated by the human body, with minimal adverse reactions, making it ideal for use in implants and prosthetic applications. Despite these advantages, Ti6Al7Nb is bioinert, which limits its biological integration and mechanical stability. Bioinert surfaces do not provide the necessary cues for cells to adhere, spread, and differentiate effectively, potentially leading to slower or incomplete healing and osteointegration. Without proper osseointegration, the implant cannot bond with the surrounding bone, increasing the likelihood of failure. For instance, in joint implants, suboptimal osteointegration can result in the release of wear particles, potentially causing inflammation and osteolysis.^4^

One promising strategy to improve the biological response of bare metal surfaces is to create nanostructured topographies.^5^ Anodic oxidation (anodization) is a simple and cost-effective method for producing nanostructured features on titanium (Ti) alloys. This technique enables the formation of self-organized, highly ordered, and well-aligned nanotube structures on Ti alloys.^6,7^ The length, pore size, roughness and wall thickness of these nanotube can be precisely controlled by altering electrochemical parameters, providing an opportunity to optimize the surface features for biomedical applications.^8,9^ Several studies have highlighted the importance of optimizing the pore size of nanotubes for bioactivity and osteointegration, as the pore size significantly influences cellular response.^10,11^ Optimal pore sizes can promote cellular attachment, proliferation, and differentiation, which are essential for successful osseointegration.^12,13^ However, if the pores are too small, cells may not adhere properly, while excessively large pores might not support effective cell bridging and tissue formation.^14^

In addition to biological interactions, implants are exposed to corrosive environments due to bodily fluids and the acidic environment that arises postimplantation. An oxide layer can serve as a protective barrier, preventing the underlying metal from corroding and thereby prolonging the implant’s lifespan Although Ti6Al7Nb has high corrosion resistance, it remains susceptible to corrosion under the harsh physiological conditions within the body. Anodized nanotubes can be particularly beneficial in this regard, as the thickness and structure of the oxide layer can significantly influence corrosion resistance. For instance, longer nanotubes, which form a thicker oxide layer, may offer better corrosion protection. However, they can be more prone to mechanical failure under mechanical stress.^14,15^ In contrast, short tubes form a more compact oxide layer with stronger interfacial bonding, potentially enhancing mechanical stability. Park et al. demonstrated that the critical load for delamination during scratch testing is significantly higher in short nanotubes than in longer ones, indicating better adhesion onto the underlying substrate.^16^ Another study investigating the effect of anodization duration on nanotube adhesion strength reported critical loads of 12 mN, 20 mN, 47 mN, and 24 mN for samples anodized for 20, 40, 50, and 60 min, respectively, at 60 V inside a NH_4_F electrolyte, indicating that anodization duration affect adhesion strength of the nanotubes.^17^

Given that both the thickness of the anodized oxide layer and the pore size can influence the integration of implants with juxtaposed tissues, achieving an optimal balance between these factors can enhance corrosion resistance, and increase the ability to withstand mechanical stresses during implantation and subsequent biomechanical loads, while also promoting bone cell functions. In the present work, we evaluate the effects of both nanotube size and oxide layer thickness on adhesion strength, corrosion behavior, and bone cell interactions for the first time in the literature. To achieve this, we maintained a constant nanotube length of either ∼1 μm or ∼4 μm, and systematically varied the nanotube diameters formed on Ti6Al7Nb via the anodization method. This approach aims to provide a comprehensive understanding of how changes in nanotube feature size influence the overall performance of anodized Ti6Al7Nb as an orthopedic implant.

## Materials and Methods

Ti6Al7Nb rods (ASTM F1295, 18 mm diameter) were mechanically cut into disks with a thickness of 1 mm. The samples were sequentially sonicated in acetone, ethanol, and ultrapure water for 15 min each to clean their surfaces. To fabricate nanotubes on the Ti6Al7Nb disks, samples were anodized. During anodization, a platinum mesh (Alfa Aesar) served as the cathode, while the cleaned Ti6Al7Nb samples acted as the anode. Both electrodes were connected to a DC power supply (Genesys 300 V/5A, TDK Lambda). Anodization was performed in an electrolyte solution containing 0.7 wt % NH_4_F and 2.5 M H_3_PO_4_ in ethylene glycol. For the formation of approximately 1 μm thick oxide layers, anodization was performed at 10, 25, and 40 V for 210, 60, and 40 min, respectively. For the formation of approximately 4 μm thick oxide layers, the samples were anodized at 10, 25, and 40 V for 720, 300, and 240 min, respectively. The selection of these voltages and durations was primarily aimed at achieving reliable control over nanotube length and obtaining uniform nanotube sizes, while also ensuring consistent thickness across different anodization conditions and preventing the formation of surface cracks.

The surface morphology of the samples was analyzed using a scanning electron microscope (SEM, FEI Nova Nano 430). Images of nanotube bottoms and cross sections were obtained from mechanically scratched surfaces. For each sample group, measurements were carried out on at least 50 individual nanotubes, with five independent measurements per sample, using ImageJ software (NIH). Surface roughness at the nanoscale was characterized using an atomic force microscope (AFM, Veeco Multimode V). Scans were conducted over 0.9 × 0.9 μm^2^ areas at a scan rate of 1 Hz. The root-mean-square roughness (*R*_q_) and surface area were calculated using Image Plus software. Water contact angle measurements were performed using a goniometer (EasyDrop, KRÜSS GmbH, Germany). An 8 μL droplet of distilled water was placed on the sample surface, and all measurements were performed in triplicate for reproducibility. To evaluate the mechanical integrity of the oxide layer formed on the anodized samples, scratch testing was performed using a MicroScratch Tester (Anton Paar MST^3^). A progressive load was applied using a Berkovich-type indenter with a 5 μm tip diameter, starting from 0.05 N and increasing at a rate of 0.02 N/min up to a maximum load of 0.2 N. Each scratch was performed over a 1 mm-long track on the sample surface. The tests were repeated five times to ensure reproducibility. The chemical composition of the nonanodized and nanostructured Ti6Al7Nb surfaces was analyzed by X-ray photoelectron spectroscopy (PHI 5000 VersaProbe) using a monochromatic Al Kα source. Wide scan spectra and high-resolution spectra for Ti 2p, Nb 3d, Al 2p and O 1s were collected. Prior to high resolution analysis, surfaces were cleaned with Ar^+^ sputtering (1 keV, 3 min).

The electrochemical properties of the samples were evaluated using a three-electrode potentiostat system (Gamry, Interface 1010). Ti6Al7Nb samples were used as the working electrode, a silver/silver chloride (Ag/AgCl) electrode served as the reference electrode, and a platinum (Pt) electrode was used as the counter electrode. Prior to testing, the open circuit potential (OCP) was monitored for 2 h in 1× phosphate-buffered saline (1× PBS) to stabilize the system. Electrochemical impedance spectroscopy (EIS) was conducted by applying a 10 mV AC signal over a frequency range of 100 kHz to 1 MHz. Tafel extrapolation was performed to determine the corrosion potential (*E*_corr_) and corrosion current density (*I*_corr_). The resulting data were analyzed using Gamry software.

The *in vitro* biological performance of the surfaces was evaluated using a human osteoblast cell line (hFOB 1.19, ATCC CRL-11372). The cells were cultured in Dulbecco’s Modified Eagle Medium (DMEM) supplemented with 10% fetal bovine serum (FBS), 2 mM l-glutamine, and 1% penicillin/streptomycin (P/S) under standard cell culture conditions (37 °C, 5% CO_2_).^18^ Cell proliferation on the samples was assessed using the 3-(4,5-dimethylthiazol-2-yl)-2,5-diphenyltetrazolium bromide (MTT) assay. hFOB cells were seeded onto the sample surfaces at a density of 2 × 10^4^ cells/cm^2^ and cultured for up to 5 days under standard conditions. At designated time points, the samples were rinsed with 1× PBS and transferred to fresh wells. Then, 1 mL of MTT solution (1 mg/mL) was added to each sample, and the samples were incubated for 4 h to allow the formation of formazan crystals. The resulting formazan was solubilized using 0.1 M HCl prepared in isopropanol. Subsequently, 200 μL of the dissolved solution from each sample was measurement at 570 nm using a microplate reader (Thermo Scientific Multiskan Go). Absorbance values obtained from cell-free (blank) samples were subtracted from all readings. The MTT assay was performed in triplicate, with three samples per replicate. Cellular morphology was evaluated using immunofluorescence imaging. hFOB cells were seeded onto sterile samples and cultured under standard cell culture conditions (37 °C, 5% CO_2_). Prior to imaging, the cells were fixed, permeabilized with 0.2% Triton X-100 for 30 min and blocked with 5% bovine serum albumin (BSA) for 30 min. Actin filaments were stained using phalloidin (1:200 dilution) for 1 h. Cell nuclei were counterstained with 4′,6-diamidino-2-phenylindole dihydrochloride (DAPI) solution (1:40,000 dilution) for 30 min. Fluorescence images were acquired using a confocal microscope (Zeiss LSM800).

Statistical analysis was performed using SPSS software (Armonk, NY). One-way analysis of variance (ANOVA) followed by Tukey’s post hoc test was used to evaluate differences between groups. Results are presented as mean ± standard error of the mean (SEM). Statistical significance was defined as *p < 0.05, and **p < 0.01.

## Results and Discussion

One of the main objectives of this study was to investigate the precise control of nanotube diameter and length. Figure S1 (see Supporting Information) shows the SEM image of the NA surface (control) prior to anodization, while Figure 1 presents SEM images of the anodized samples, all exhibiting a fixed nanotube length of approximately 1 μm (S). In this study, nanotube length was optimized by adjusting the anodization duration. Meanwhile, samples were anodized at 10 V, 25 V, and 40 V, and are referred to as NT10-S, NT25-S, and NT40-S, respectively (Figure 1a–c). Both the applied voltage and anodization time influenced the formation of the nanotubes on the Ti6Al7Nb samples. Upon the anodization, nanotubes with varying pore diameters were successfully fabricated, measuring 24  ±  2 nm, 48 ±  6 nm, and 81  ±  8 nm for NT10-S, NT25-S, and NT40-S, respectively. The results clearly show that the pore diameter increased with increasing anodization voltage. Additionally, the bottom diameters of the nanotubes also increased with voltage, measured as 41  ±  6 nm, 89  ±  7 nm, and 122  ±  10 nm for NT10-S, NT25-S, and NT40-S, respectively (Figure 1d,e).

**Figure 1:**
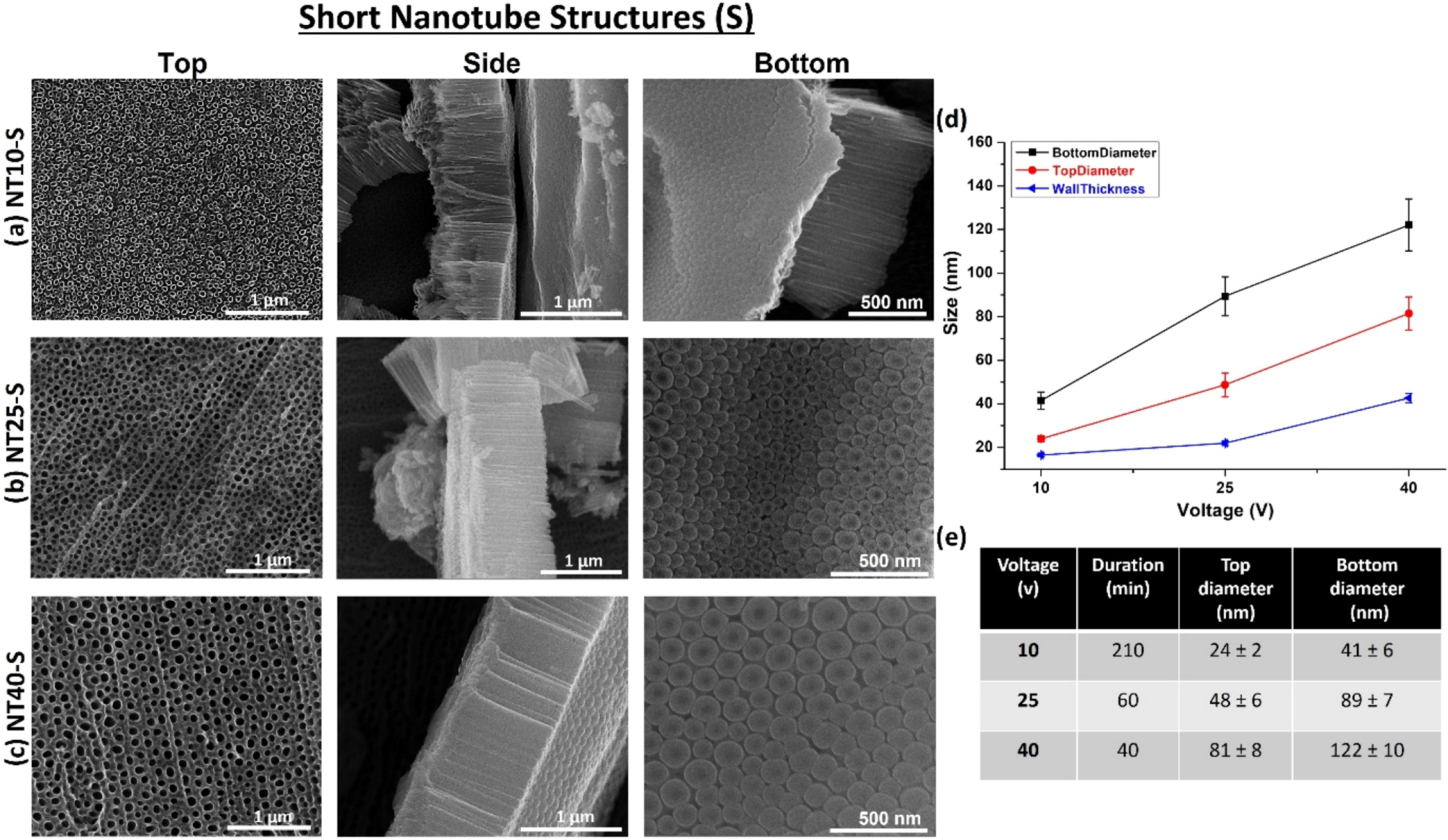
(a–c) Top (left), side (middle), and bottom (right) views of short (∼1 μm; S) nanotubes fabricated at (a) 10 V, (b) 25 V, and (c) 40 V. (d) Relationship between the applied voltage and nanotube dimensions, including top diameter, bottom diameter, and wall thickness. (e) Table summarizing the anodization voltage, duration, and corresponding top and bottom diameters of the nanotubes.

While Figure 1 presents SEM images of the samples with a fixed nanotube length of ∼1 μm, Figure 2 displays those with longer nanotube lengths (∼4 μm; L), achieved by increasing the anodization duration. Nanotubes with top diameters of 22  ±  2 nm, 60  ±  5 nm, and 97  ±  6 nm were successfully fabricated for NT10-L, NT25-L, and NT40-L, respectively (Figure 2a–c). The corresponding bottom diameters were measured as 44  ±  3 nm, 108 ±  9 nm, and 127  ±  8 nm for NT10-L, NT25-L, and NT40-L, respectively. These results demonstrate that anodization parameters—particularly voltage and duration—have a significant influence on nanotube formation, which can, in turn, affect surface properties. As shown in Figures 1d and 2d, the pore diameter of the nanotubes on Ti6Al7Nb surfaces is strongly dependent on the applied voltage. However, anodization duration also played a role in modulating nanotube diameter at higher voltages. At a lower voltage (10 V), increasing the anodization duration from 210 to 720 min did not alter the nanotube diameter (*p* > 0.05). In contrast, at a higher voltage (40 V), increasing the duration from 40 to 240 min resulted in a ∼15% increase in nanotube diameter (from 81 nm to 97 nm). Although the mechanism behind the nanotube diameter change is not fully clear, it can be speculated that lower voltages may not provide sufficient driving force for field-assisted/chemical etching to widen the nanotubes over the tested anodization durations. On the other hand, oxide layer thickness (i.e., nanotube length) was primarily governed by anodization duration. Specifically, to obtain nanotubes of 1 μm and 4 μm in length, anodization durations of 210 and 720 min at 10 V, 60 and 300 min at 25 V, and 40 and 240 min at 40 V were required, respectively. A more comprehensive analysis of nanotube length variation at 40 V with different anodization durations was shown in Supporting Figure S2. This figure reveals that nanotube length increases with anodization duration. Moreover, both for 1 μm and 4 μm long nanotubes, there was a difference between the bottom and top diameters, as shown in Figures 1e and 2e. This difference can be attributed to the slightly tapered morphology of the nanotubes, which can arise from the interplay between field-assisted oxidation and localized chemical dissolution processes potentially promoting more rapid oxide dissolution at the tube base.^19,20^

**Figure 2:**
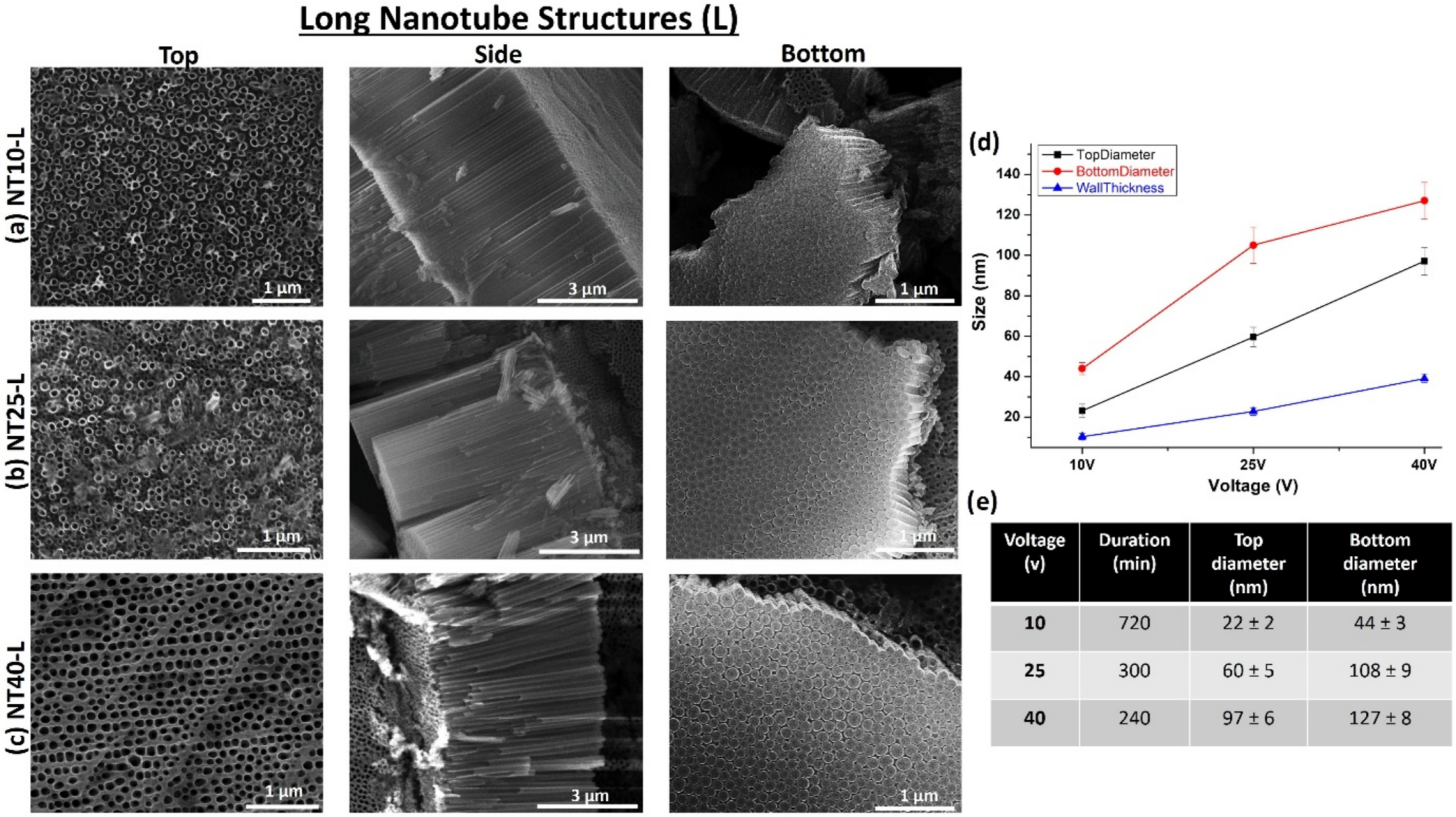
(a–c) Top (left), side (middle), and bottom (right) views of long (∼4 μm; L) nanotubes fabricated at (a) 10 V, (b) 25 V, and (c) 40 V. (d) Relationship between the applied voltage and nanotube dimensions, including top diameter, bottom diameter, and wall thickness. (e) Table summarizing the anodization voltage, duration, and corresponding top and bottom diameters of the nanotubes.

Potentiodynamic polarization experiments were conducted in 1× PBS to evaluate the corrosion behavior of the samples. Figure 3 presents the Tafel plots of NA and anodized nanostructured samples. The corrosion potential (*E*_corr_) and corrosion current density (*I*_corr_) were determined using Tafel fitting. The corrosion potential of the NA sample was measured as −0.28 V. For samples with 1 μm-thick nanotubes, *E*_corr_ values were −0.23 V for NT10-S and −0.35 V for NT40-S. The samples with 4 μm-thick nanotubes exhibited *E*_corr_ values of −0.22 V for NT10-L and −0.44 V for NT40-L. The lowest corrosion current (*I*_corr_) was observed for the NT10-S sample at 203.9 nA, while the highest value was found for the NT40-L sample at 1620.0 nA. For comparison, *I*_corr_ values for NT40-S and NT10-L were calculated to be 691.2 nA and 397.6 nA, respectively. Electrochemical impedance spectroscopy (EIS) is a widely used technique to study electrochemical properties of porous materials, providing insight into both the internal resistance of the surface and the interfacial resistance between the surface and the electrolyte. Supporting Figures S3 and S4 compares the Bode and Nyquist plots of the NA and nanotube samples. The results showed that *R*_ct_ value of the NT10-S sample was the highest, while the corresponding *C*_dl_ value was the lowest (Table S1). The high *R*_ct_ value indicated the superior role of the NT10-S oxide layer in limiting the diffusion of corrosive ions into the pores of the sample, while the low *C*_dl_ suggests a reduced effective electrochemical surface area. Together, these results imply that the oxide layer effectively hindered electron transfer processes. The EIS results further demonstrate that the electrochemical stability of the nanotube surface is dependent on the nanotube diameter. As the diameter increased, corrosion resistance was observed to decrease. Similarly, Liu et al. reported that increasing nanotube diameters from 22 to 86 nm, reduced the electrochemical stability of anodized surfaces.^21^ This effect has been attributed to changes in capacitance behavior, which are governed by both the effective surface area and the presence of nonstoichiometric defects such as oxygen vacancies and titanium interstitials. With decreasing pore diameter, the surface area-to-volume ratio increases, influencing these defect populations and thereby affecting the electrochemical response. Likewise, Anitha et al. demonstrated that smaller nanotube diameters exhibited higher charge conductivity and superior electrochemical performance.^22^ These findings show that nanotube diameter, and nanotube length significantly influence the corrosion behavior of the samples. The corrosion rate increased with an increase in both oxide layer thickness and nanotube diameter. The increased corrosion rate observed for samples with longer nanotubes may be attributed to several factors. First, longer nanotubes inherently provide a greater surface area exposed to the electrolyte (1× PBS), which can enhance electrochemical activity, and thus may increase the corrosion rate. Additionally, anodized nanotubes are typically more hydrophilic,^23,24^ promoting more efficient wetting and electrolyte penetration into the nanotube arrays. This enhanced electrolyte accessibility may facilitate ionic transport and accelerate electrochemical reactions at the metal–electrolyte interface.^25^ Moreover, the increased depth of the nanotubes could lead to slower diffusion of corrosion products out of the tubes and hinder oxygen replenishment inside them, creating microenvironments that locally shift the pH or redox potential—conditions known to promote corrosion.^26^ However, this remains speculative and would require further investigation through detailed localized surface analysis, such as cyclic polarization or scanning electrochemical microscopy.^27^

**Figure 3:**
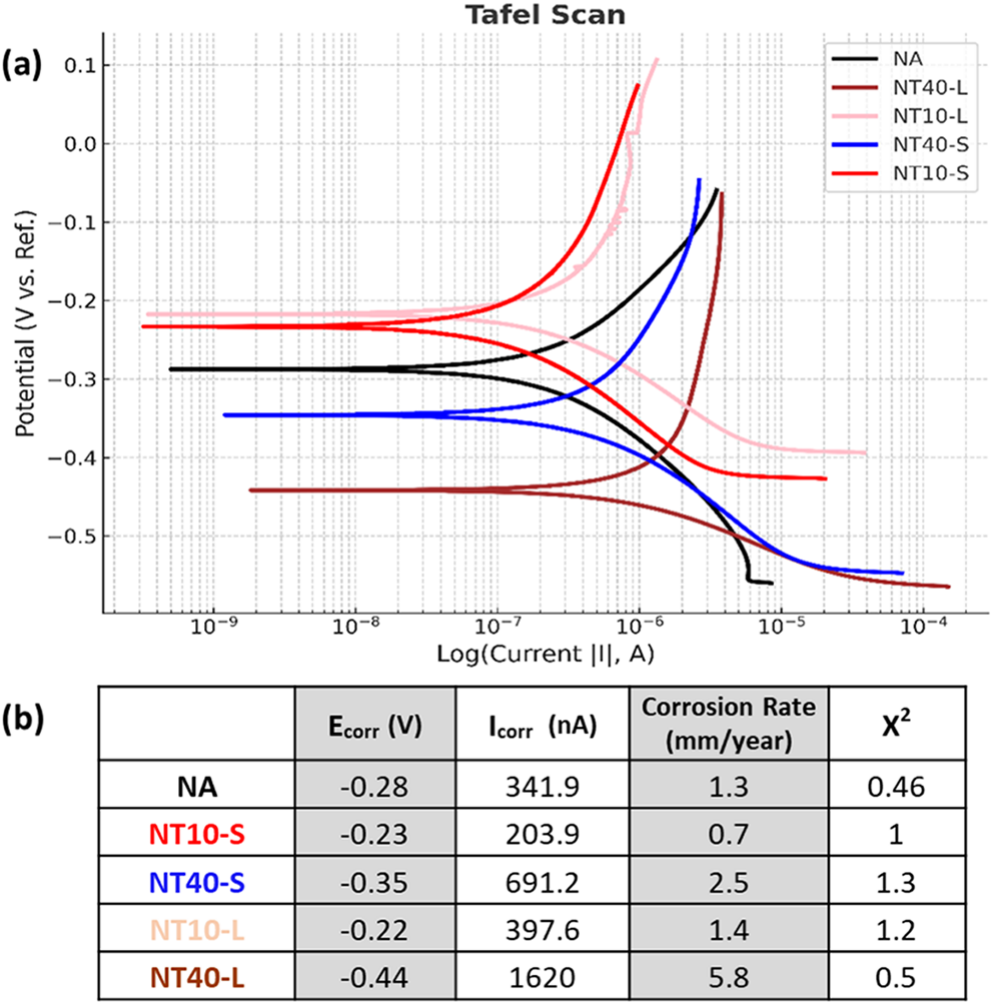
-(a) Tafel plots of NA, NT10-S, NT40-S, NT10-S, and NT10-L samples. The length and diameter of nanotubes influence corrosion behavior. Especially, smaller diameter nanotubes inhibit ionic transfer compared to larger diameter. (b) Table summarizing the *E*_corr_, *I*_corr_ and corrosion rates and chi square values of the samples.

Oxide layers with two distinct nanotube lengths—approximately 1 μm and 4 μm—were initially fabricated to investigate the effect of nanotube dimensions on material performance. However, corrosion testing revealed that surfaces having 4 μm-thick nanotubes exhibited lower corrosion resistance compared to their shorter counterparts, which is undesirable for orthopedic applications. In addition to their superior corrosion behavior, shorter nanotubes have also been reported to offer better mechanical stability. Hu et al. found that short nanotubes exhibit stronger adhesion to the titanium substrate, while longer nanotubes are more prone to delamination due to increased interfacial stress.^28^ Similar observations were made by Cao et al., who reported weaker adhesion in thicker nanotube layers.^29^ Based on these findings, we infer that short nanotubes (∼1 μm) provide better mechanical stability, resulting in a more compact and robust oxide layer that is less prone to failure under external stress. In addition to this mechanical advantage, as previously discussed, the 1 μm-thick nanotubes also exhibited superior corrosion resistance. Therefore, subsequent mechanical and biological evaluations were focused on samples with 1 μm nanotube lengths.

The mechanical integrity of the nanostructured Ti6Al7Nb surface, particularly the adhesion strength of the nanotube layer, is critical for the long-term performance of load-bearing orthopedic implants. Figure 4 presents the results of the microscratch tests and Supporting Figure S6 shows images of the scratched surfaces. In these profiles, the red, purple, and brown lines correspond to the coefficient of friction, frictional force, and applied normal force, respectively. The critical loads, defined as the point at which coating failure initiates, were measured as 0.16 ± 0.02, 0.13 ± 0.01 and 0.11 ± 0.01 N for NT10-S, NT25-S, and NT40-S respectively. Fluctuations observed in the force signals suggest localized cracking or damage to the nanotubes during the scratch process. Among all samples, NT10-S exhibited the highest critical load, indicating superior adhesion and mechanical stability. Since all samples had comparable oxide layer thicknesses (∼1 μm), the observed differences in mechanical performance are likely attributed to variations in nanotube diameter. Smaller-diameter nanotubes are generally more densely packed, which has been associated with enhanced adhesion strength and improved mechanical integrity of the oxide layer.^17^ This densely packed nature is clearly visible in the bottom images of the nanotubes (Figures 1 and 2). In addition to packing density, surface roughness has also been proposed as a critical factor influencing both the adhesion strength and the failure mechanisms of TiO_2_ nanotube layers.^30^ Increased nanostructured roughness can enhance mechanical interlocking and reduce the likelihood of delamination by redistributing residual stresses across the coating. Based on these findings, the superior mechanical performance observed for the NT10-S sample may be attributed to the synergistic effect of higher nanotube packing density and increased surface roughness when compared to the NT40-S sample.

**Figure 4:**
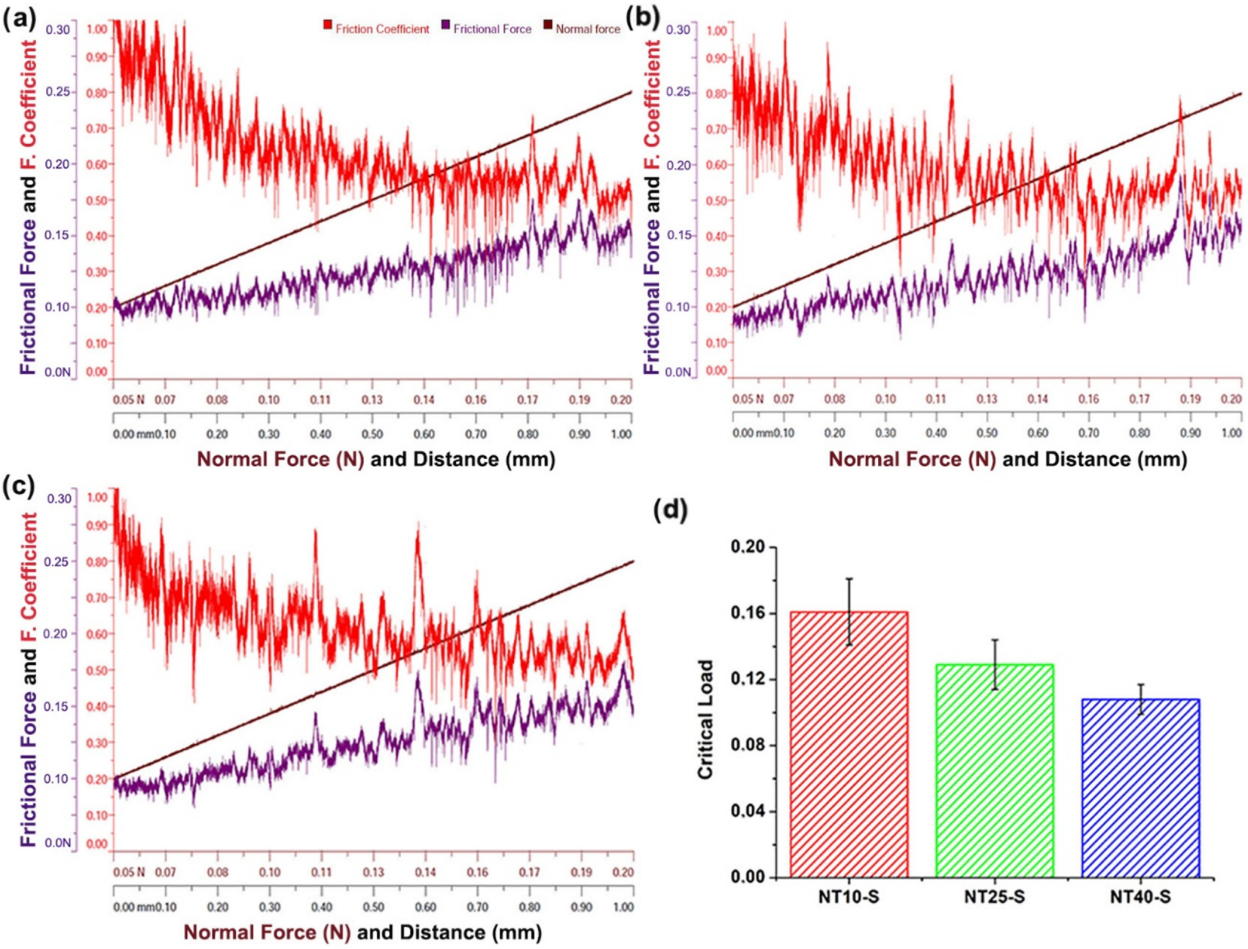
(a–c) Normal force (brown), frictional force (purple) and friction coefficient (red) curves obtained during the microscratch testing on the (a) NT10-S, (b) NT25-S, and (c) NT40-S samples. (d) Critical load values for each sample.

Figure 5 presents 3D AFM micrographs, showing that anodization process successfully created distinct nanostructured topographies on the Ti6Al7Nb surfaces. AFM analysis revealed that root-mean-square surface roughness (*S*_q_) values were 2.9  ±  0.4 nm for the NA surface, and 21.2  ±  2.4 nm, 10.3  ±  0.8 nm, and 14.8  ± 1.9 nm for the NT10-S, NT25-S, and NT40-S samples, respectively. These results clearly demonstrate that the anodization process significantly increased the nanostructured surface roughness compared to NA. In addition to increased roughness, the surface area of the samples also increased upon anodization, enhancing the surface’s potential for cellular interaction. The surface area values were measured as 0.8  ±  0.1 μm^2^ for NA, 1.5 ±  0.2 μm^2^ for NT10-S, 1.1  ± 0.1 μm^2^ for NT25-S, and 1.3  ± 0.2 μm^2^ for NT40-S. Notably, the NT10-S sample—with the smallest pore size—exhibited the highest surface roughness and surface area among all anodized samples.

**Figure 5:**
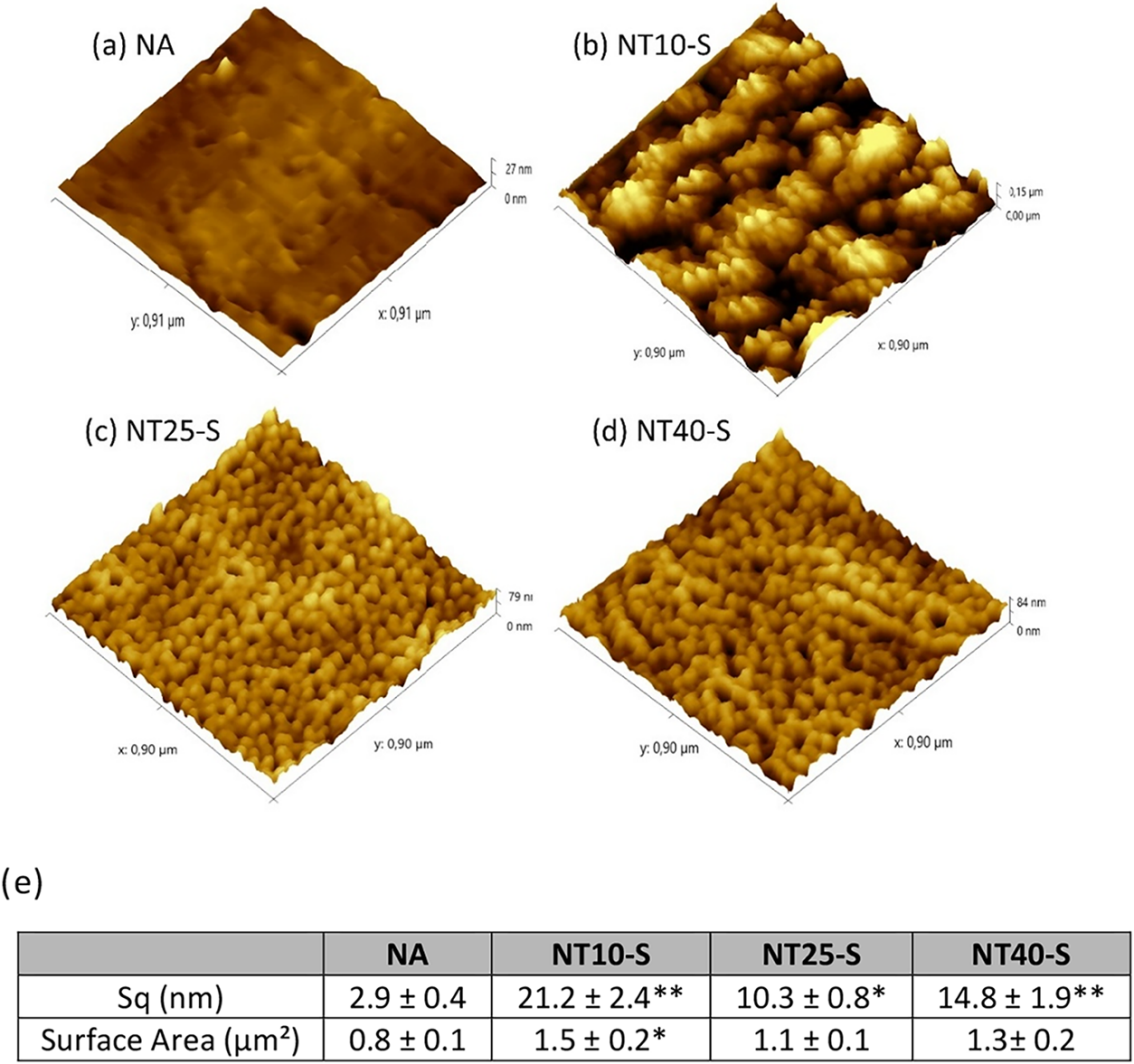
3D AFM images of (a) NA, (b) NT10-S, (c) NT25-S, and (d) NT40-S surfaces. (e) Surface roughness values and surface areas of the samples (*p < 0.05, and **p < 0.01 compared to NA, values are mean ± SD, *n* = 3).

The wide scan and high-resolution XPS spectra for the NA, NT10-S, and NT40-S samples are shown in Figure 6. The wide scan spectra indicated that all surfaces contained C, O, N, Ti, Al, and Nb.^31^ The carbon and nitrogen signals were attributed to adventitious hydrocarbons and airborne contamination, respectively. Oxygen, on the other hand, originated from the native oxide and anodic oxide films for the nonanodized and anodized samples, respective. On the nanostructured sample surfaces, fluorine (F) and phosphorus (P) were also detected, consistent with residual electrolyte species. On the other hand, the NA surface exhibits silicon (Si), likely introduced during the sample preparation steps, and disappeared upon the anodization.

**Figure 6:**
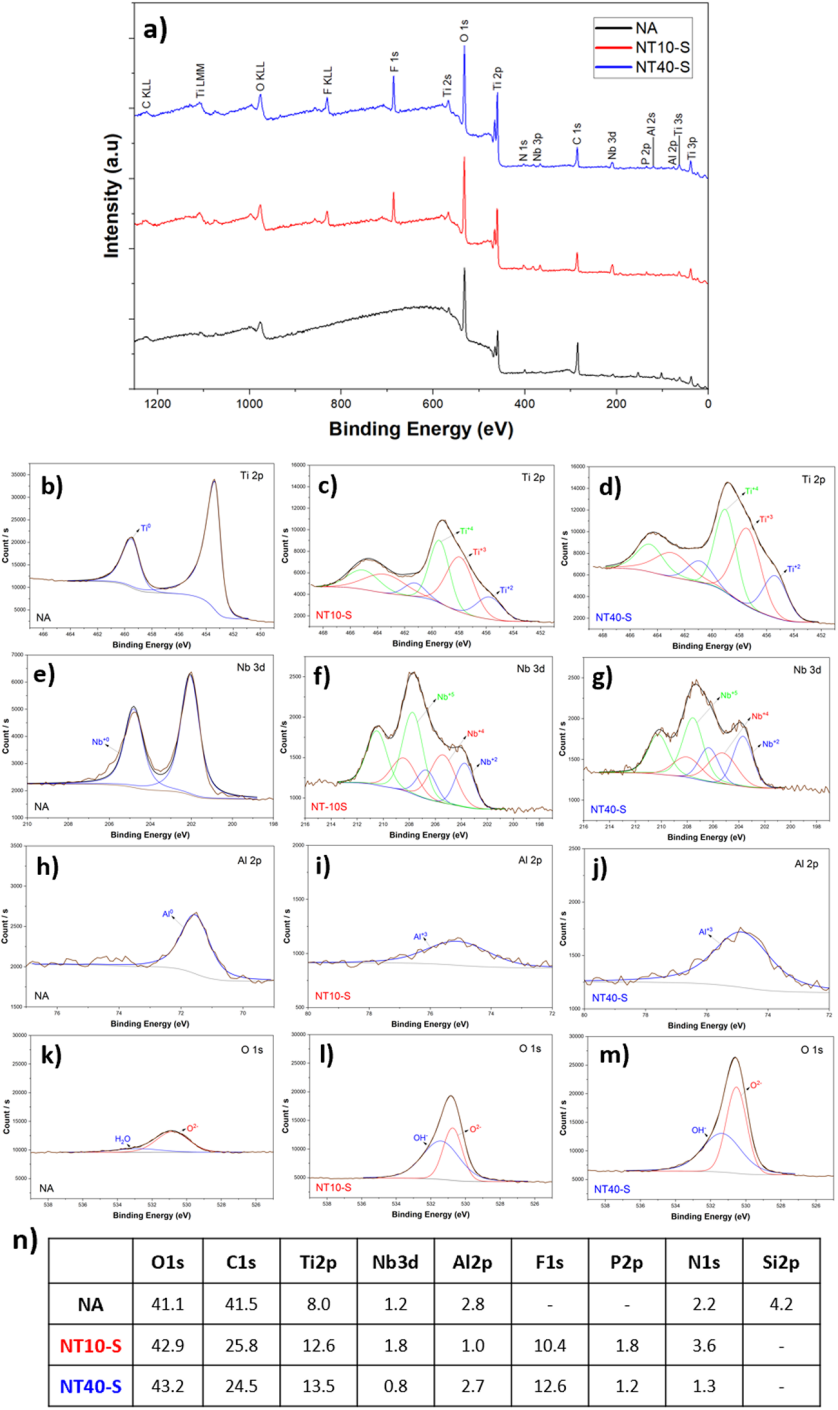
(a) Wide scan XPS spectra of the NA, NT10-S, and NT40-S samples obtained from the nonsputtered surfaces. (b–m) High resolution (b–d) Ti 2p, (e–g) Nb 3d, (h–j) Al 2p, and (k–m) O 1s spectra of (b, e, h, k) NA, (k, f, i, l) NT-10S, and (d, g, j, m) NT-40S samples obtained from the sputtered surfaces. (n) Compositional analysis of the sample surfaces obtained from the wide scan spectra. Values are given in atom %.

High resolution XPS spectra for the Ti 2p are displayed in Figure 6b–d. For the NA sample, the high-resolution Ti 2p spectrum showed a well-resolved metallic doublet at 453.5 eV (2p_3/2_) and 459.6 eV (2p_1/2_), giving a spin–orbit separation of 6.1 eV, fully consistent with Ti^0^.^31,32^ For the NT10-S sample, Ti 2p_3/2_ spin orbit peaks for Ti^4+^, Ti^3+^ and Ti^2+^ appeared at 459.5, 458.0, and 455.8 eV, respectively. For NT40-S, the Ti^4+^, Ti^3+^ and Ti^2+^ peaks shifted to 459.1, 457.5, and 455.5 eV, respectively.^33^ Aside from Ti 2p, high resolution XPS spectra for the Nb 3d are displayed in Figure 6e–g. For the NA sample, Nb 3d spectrum exhibited a single doublet at 202.1 eV (3d_5/2_), corresponding to metallic Nb^0^. In contrast, the anodized samples displayed multiple oxide states. The NT10-S sample exhibited Nb 3d_5/2_ peaks positioned at 207.7, 205.5, and 203.8 eV, which were attributed to Nb^5+^ Nb^4+^ and Nb^2+^, respectively. Similarly, for the NT40-S sample, Nb 3d_5/2_ peaks appeared at 207.6, 205.3, and 203.7 eV, corresponding to the same oxidation states.^34^ In addition, Al 2p core level analysis is displayed in Figure 6 h-j. For the NA sample, high resolution spectra of Al 2p showed only metallic Al (Al^0^) peak at 71.5 eV, while Al^3+^ peaks were observed at 75.2 and 74.9 eV for NT10-S and NT40-S, respectively.^35^ For the Ti 2p, Nb 3d, and Al 2p core levels, a slight shift toward lower binding energies was observed for NT40-S compared to NT10-S. During anodization, oxygen anions (O^2–^/O^–^) migrate through the already-formed oxide layer toward the metal/oxide interface. Across the oxide film layer, the electrical field drops, gradually slowing anion transport (field-assisted migration).^36^ Consequently, the oxygen concentration varies with depth, favoring the formation of oxides with various valancies (specifically for Ti and Nb) within the anodic layer. High-resolution spectra of the O 1s regions of the samples are given in Figure 6k–m. The NA sample exhibited adsorbed H_2_O peak at 533.0 eV and lattice O^2–^ peak at 530.8 eV. It should be noted that the intensity of the O^2–^ peak was very low for the NA samples, which was in-line with the absence of oxide peaks on the Ti 2p, Nb 3d and Al 2p spectra of the NA sample. On the other hand, the O 1s spectra of NT10-S and NT40-S confirm the presence of oxygen in the anodic films. For NT10-S, peaks appeared at 530.8 eV (O^2–^) and 531.5 eV (−OH); and for NT40-S, they appear at slightly lower eVs, 530.5 and 531.4 eV, respectively. The O^2–^ peaks are attributed to metal oxides, whereas the −OH component arises from hydroxyl species diffusing into the anodic layer.^37^

The change in the chemistry of the nanotubes, aside from increased length and improved hydrophilicity, can potentially provide an explanation for the observed corrosion behavior, particularly the lowest corrosion rate of NT10-S. It is established that the chemistry of the surface oxide layers, specifically Nb and F contents, can alter the corrosion resistance of materials. According to the XPS analysis, the Nb contents were determined as 1.2, 1.8, and 0.8 atom % for the NA, NT10-S, and NT40-S samples, respectively (Figure 6n). The surface Nb concentration correlated with the corrosion potential, such that as the Nb amount increased, the corrosion potential became less negative, indicating a more noble surface. Increasing Nb content on the outermost surface of the samples shifted the corrosion potential to more positive values, potentially due to the wider band gap of Nb_2_O_5_ compared to TiO_2_. This would suppress electron transfer across the anodic film and metal/electrolyte interface.^38^ In addition, Nb_2_O_5_ has a more negative standard enthalpy of formation than TiO_2_, indicating a more thermodynamically stable oxide.^39^ Aside from the influence of Nb, the F content of the NT40-S surfaces was observed to be higher than the one for the NT10-S sample. F^–^ ions in the oxide layer form fluoride complexes (i.e., TiF_6_^2–^), which could potentially shift the corrosion potential toward more negative values, leading to the increase in the corrosion current with increased F^–^ content.^40,41^

The surface hydrophilicity of the samples following anodization was evaluated using water contact angle measurements. As shown in Figure 7, NA surface exhibited a contact angle of 58.9  ±  3.9°, whereas the anodized surfaces NT10-S, and NT40-S displayed significantly reduced contact angles of 25.1  ±  1.9°, and 9.8 ±  1.9°, respectively. These results indicate that surface hydrophilicity increased substantially following anodization. Water contact angles are influenced by several surface characteristics, including pore size, surface roughness, and chemical composition.^42^ XPS analysis confirmed that nanostructured surface had different oxide peaks of Ti 2p while NA surface had metallic Ti peak. Also, hydroxyl (−OH) peaks on nanostructured surface effect the hydrophilicty. Similarly, we demonstrated that surface hydroxyl groups enhance wettability by attracting water molecules through hydrogen bonding in our previous work.^43^ Additionally, increased surface roughness can improve wettability by enlarging the effective contact area between the surface and water, consistent with the Wenzel model.

**Figure 7:**
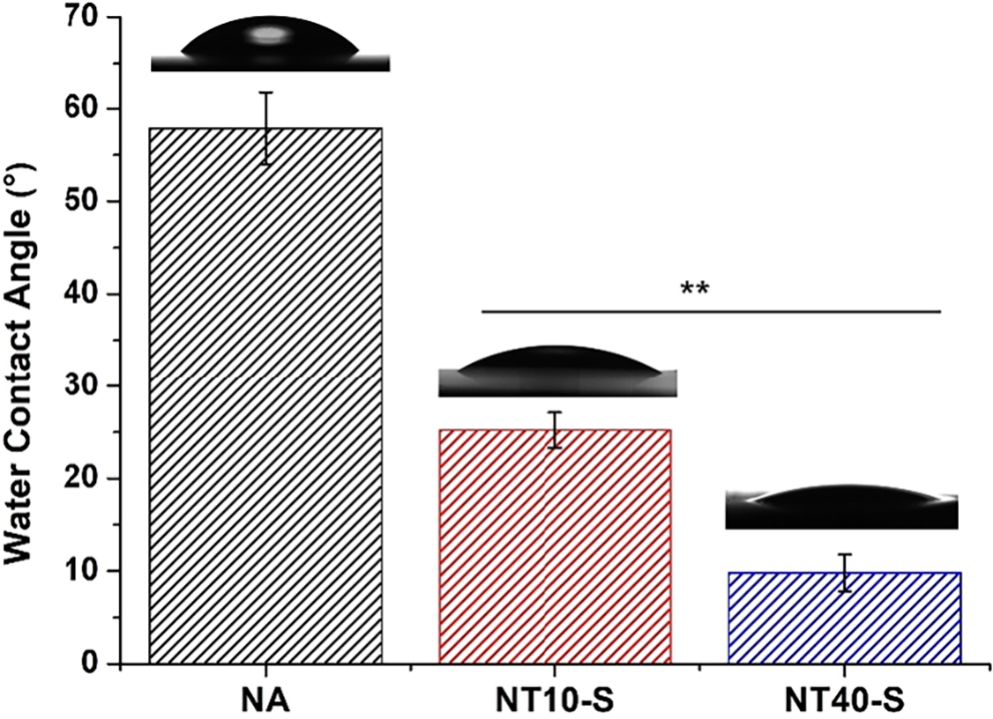
Water contact angle values of the NA, NT10-S and NT40-S samples (**p < 0.01 compared to NA, values are mean ± SD, *n* = 4). Nanotubes provide hydrophilic characteristic compared to bare sample.

Figure 8a presents the results of the MTT assay, showing osteoblast proliferation on NA, NT10-S, and NT40-S samples. The results indicate that the NT10-S significantly enhanced osteoblast proliferation compared to NA on both day 3 (*p* < 0.05) and day 5 (*p* < 0.01) of culture. Cellular morphology and cytoskeletal organization were further investigated using fluorescence staining (Figure 8b–d). The images revealed well-organized actin filaments on the NT10-S surfaces, along with visually higher cell density, confirming increased osteoblast proliferation. This improved biological response is attributed to the increased nanostructured roughness and surface area of the NT10-S samples (Figure 5), which promote better osteoblast proliferation, and viability. Previous studies have demonstrated that cellular adhesion is sensitive to nanostructured surface roughness, which can enhance integrin activation and downstream signaling pathways.^43^ In addition, the enhanced hydrophilicity of the anodized surfaces suggests potential benefits, including reduced nonspecific protein adsorption and increased adsorption of fibronectin and vitronectin—both of which contain the RGD (arginine–glycine–aspartic acid) peptide sequence known to promote osteoblast functions. The enhanced biological performance observed on the anodized surfaces in our study aligns with previous reports emphasizing the favorable cellular responses to nanoscale topographies. In particular, the improved cell adhesion, proliferation, and osteogenic differentiation observed here are in agreement with studies showing that nanotubes with diameters in the range of 15–20 nm can significantly enhance these responses.^44,45^ Additionally, the osteogenic potential of anodized nanotubes has been associated with accelerated early stage bone regeneration and improved peri-implant osseointegration.^46,47^

**Figure 8:**
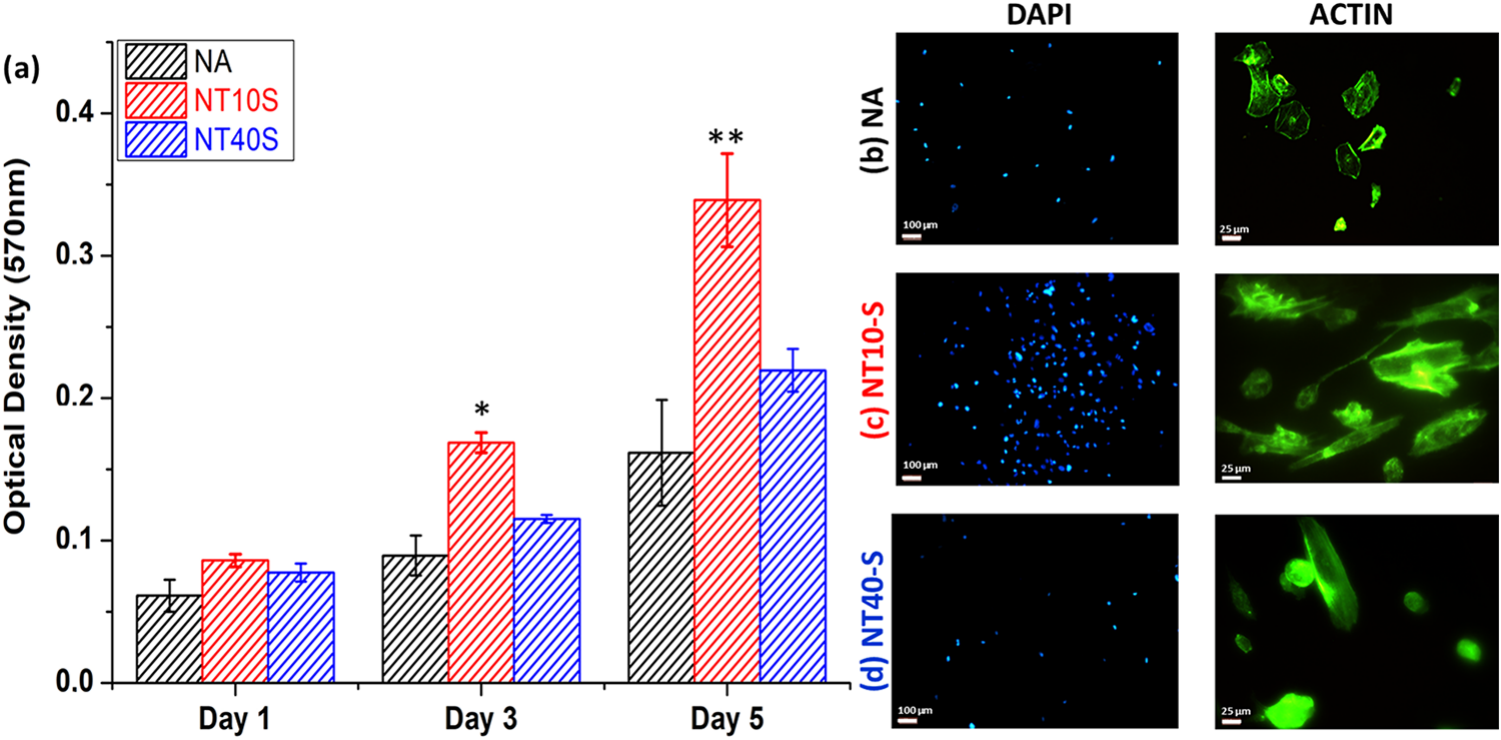
(a) Osteoblast proliferation on NA, NT10-S, and NT40-S samples over 5 days of *in vitro* culture, as assessed by the MTT assay (*p < 0.05, **p < 0.01 compared to NA, values are mean ± SD, *n* = 3). (b–d) Fluorescence images showing DAPI-stained nuclei and phalloidin-stained actin filaments of osteoblasts cultured on (b) NA, (c) NT10-S, and (d) NT40-S surfaces.

Successful osseointegration—the stable and functional connection between bone and implant—relies heavily on both mechanical interlocking and biological compatibility. Engineering a nanotube surface on titanium implants enhances mechanical stability by increasing surface roughness, which improves load distribution and promotes stronger interfacial bonding with bone tissue. When both mechanical robustness and chemical stability are achieved, nanotube surfaces can serve as effective mechanical and biological adapters at the bone–implant interface. Stable primary fixation is especially critical to minimize micromotion that could otherwise disrupt bone healing. In addition to enhancing surface area, control over the nanotube structures can also improve scratch resistance, helping preserve the nanoscale topography during implantation and under physiological loading conditions. This mechanical durability is vital for maintaining surface integrity and long-term implant performance.

Beyond mechanical benefits, the surface properties and nanotube morphology of the implant critically influence biological responses. Nanostructured topographies, by mimicking the natural extracellular matrix, facilitate protein adsorption and promote osteoblast adhesion, proliferation, and differentiation.^48^ Elongated nanotubes further provided a unique topography and enhanced effective surface area, offering more contact points for membrane receptors and thereby strengthening cell–implant interactions.^49,50^ Importantly, the creation of nanostructured topographies on implant surfaces was shown to influence cytoskeletal organization, focal adhesion formation, and intracellular signaling pathways, ultimately guiding osteoblast cellular functions and differentiation.^51^ The nanotube diameters (set by anodization voltage) can align with the dimensions of integrin clusters, facilitating focal adhesion formation and cytoskeletal organization.^52,53^ Aside from surface topography, hydrophobicity and surface chemistry are major parameters controlling cell-surface interactions. In our study, anodized nanotubes exhibited increased hydrophilicity, a key factor known to support protein-mediated cell attachment and osteogenic activity. Surface chemistry also played a significant role in cellular interactions. XPS analysis confirmed the presence of oxide peaks only for the nanostructured surfaces, indicating surface oxidation and chemical modification that enhance bioactivity and cellular proliferation. The titanium oxide layer provides hydroxylated sites, which increase wettability and promote integrin binding through adsorbed proteins.^42^ Oxide and hydroxyl groups bind water and proteins through hydrogen bonding and electrostatic forces, further supporting protein adsorption. For example, Kang et al. demonstrated that hydroxyl-modified titanium surfaces exhibited a stronger affinity for proteins due to enhanced electrostatic interactions, while their ordered hydroxyl arrangement reduced hydrogen bonding with water molecules, allowing proteins to more readily approach the surface.^54^ Moreover, anodized nanotubes can act as reservoirs for bioactive molecules while maintaining an open pore morphology conducive to nutrient exchange.^55^

Collectively, these geometric attributes—packing density, surface area, and length—obtained via anodization of Ti6Al7Nb created a multifunctional interface that supported enhanced cell–material interactions. In this context, the surfaces fabricated at 10 V—producing 1 μm-thick oxide layers with a nanotube diameter of 24 ± 2 nm—demonstrated an optimal balance of properties, including mechanical stability and osteoblast proliferation, supporting their potential use in orthopedic applications requiring robust osseointegration.

## Conclusions

Highly ordered and uniform nanotubes with lengths of ∼1 μm and ∼4 μm were successfully fabricated on Ti6Al7Nb surfaces via anodization. For each fixed length, the nanotube diameters were tailored by varying the anodization voltage (10 V, 25 V, and 40 V). The results demonstrated that both nanotube diameter and length significantly influenced osteoblast proliferation, corrosion resistance, and the adhesion strength of the oxide layer. The results showed that higher surface area and smaller pore diameter of nanotubes enhanced corrosion behavior. Also, the anodized surface with a 24 nm tubular diameter (NT10-S) exhibited the most favorable biological response, likely due to its enhanced nanostructured roughness and hydrophilicity among all samples. Moreover, this surface outperformed the other groups in terms of corrosion resistance and mechanical stability, while also promoting the highest level of osteoblast proliferation. These findings suggest that precisely engineered nanotubes on Ti6Al7Nb alloys can effectively enhance corrosion resistance and osteoblast function—both -critical factors for the long-term success of orthopedic implants.

## Supplementary Material



## Notes

SEM image of bare surface, the relationship between nanotube length and anodization duration at 40 V, Bode and Nyquist diagrams of nanostructured and bare surface, circuit parameters calculated from the EIS spectra, images of the bare and nanostructured surfaces after microscratched analysis (PDF)
