# Supplementary material for: Nanotube Size Alters Corrosion Behavior, Mechanical Properties, and Biological Interactions of Anodized Ti6Al7Nb
Source: ACS Omega. 2025 Dec 29;11(28):41137–48. doi: 10.1021/acsomega.5c07922 (PMC13393199; doi:10.1021/acsomega.5c07922)
Supplement: Supplementary file 1 [file ao5c07922_si_001.pdf]

# SUPPORTING INFORMATION

# Nanotube Size Alters Corrosion Behavior, Mechanical Properties and Biological Interactions of Anodized Ti6Al7Nb

Yasar Kemal Erdogan*[1,2], Merve Izmir[3,4], Olgu Cagan Ozonuk[3], Cem Bayram[5], Batur Ercan*[2,3,6]

[1] Department of Biomedical Engineering, Isparta University of Applied Science, 32200 Isparta, Turkey
[2] Biomedical Engineering Program, Middle East Technical University, 06800 Ankara, Turkey
[3] Department of Metallurgical and Materials Engineering, Middle East Technical University, 06800 Ankara, Turkey
[4] School of Materials Science and Engineering, Nanyang Technological University, 639798 Singapore
[5] Institute for Graduate Studies in Science and Engineering, Nanotechnology and Nanomedicine Division, Hacettepe University, 06800 Ankara, Turkey
[6] BIOMATEN, METU Center of Excellence in Biomaterials and Tissue Engineering, 06800 Ankara, Turkey

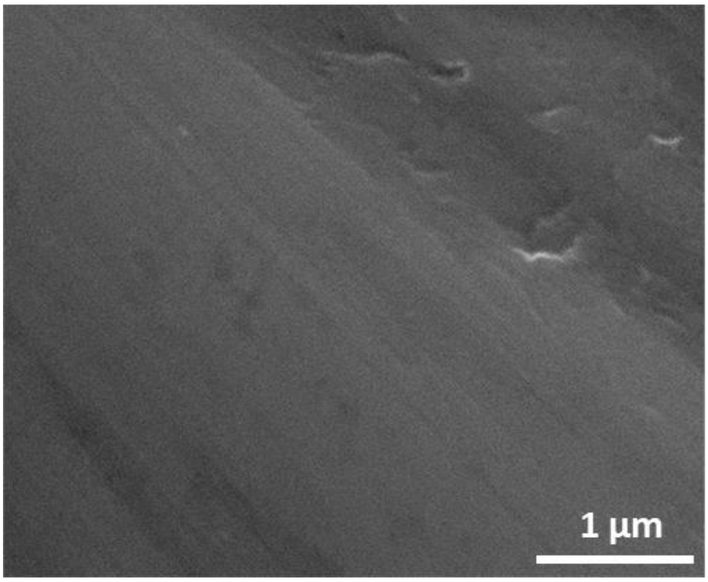


**Figure S1**. SEM image of NA (bare) surface.

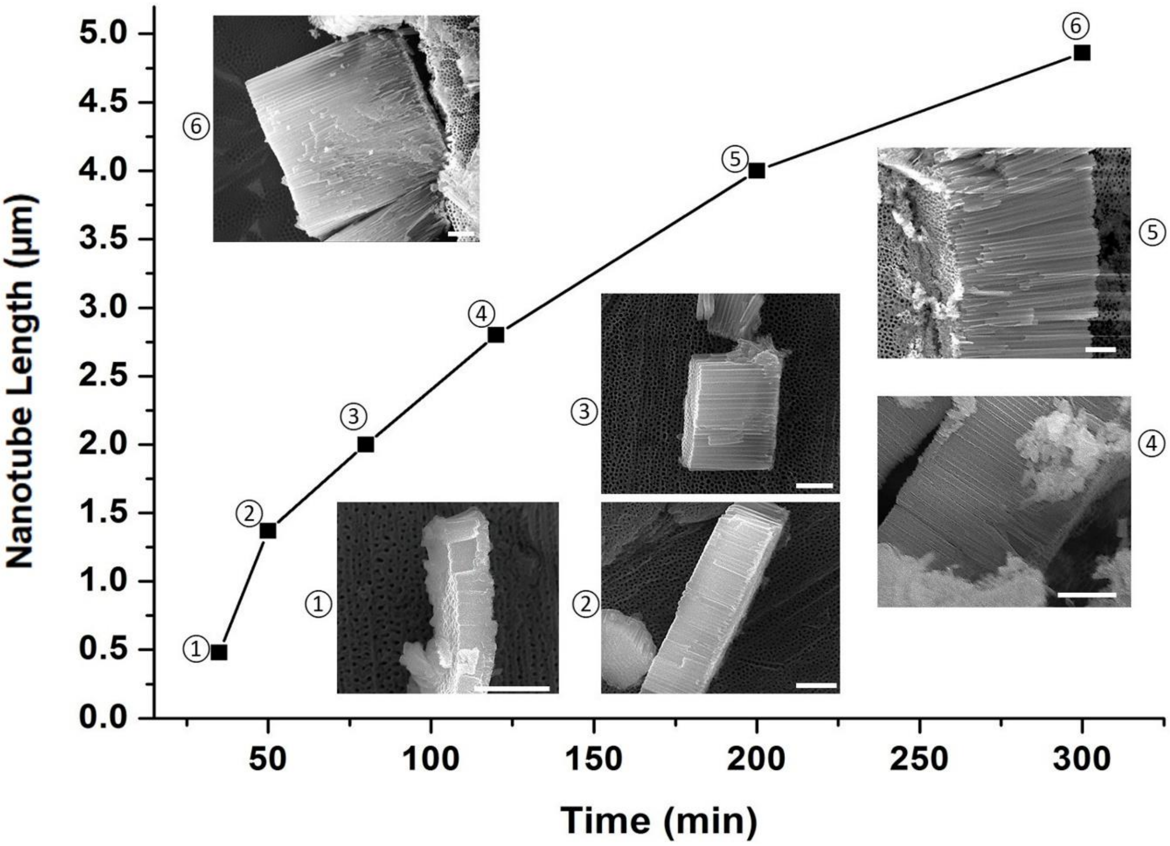


**Figure S2.** The relationship between nanotube length and anodization duration at 40V. Scale bars are 1 μm.

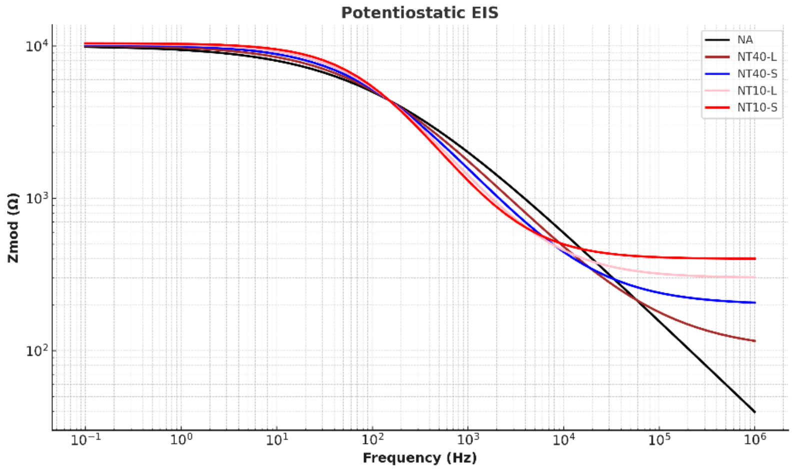


**Figure S3.** Bode diagram obtained from the EIS measurements of the samples.

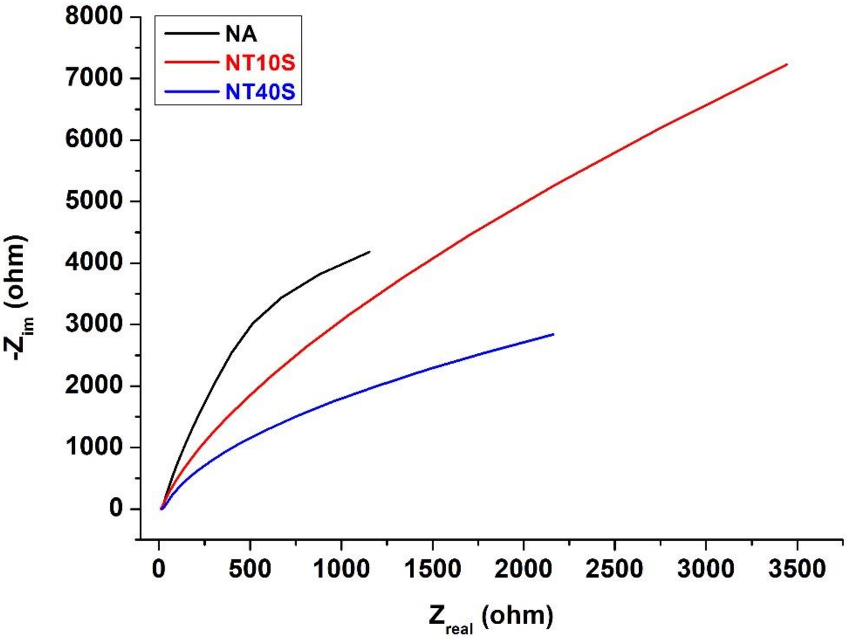


**Figure S4**. Nyquist plots of NA, NT10-S and NT40-S. The nanotube diameter influences electrochemical properties.

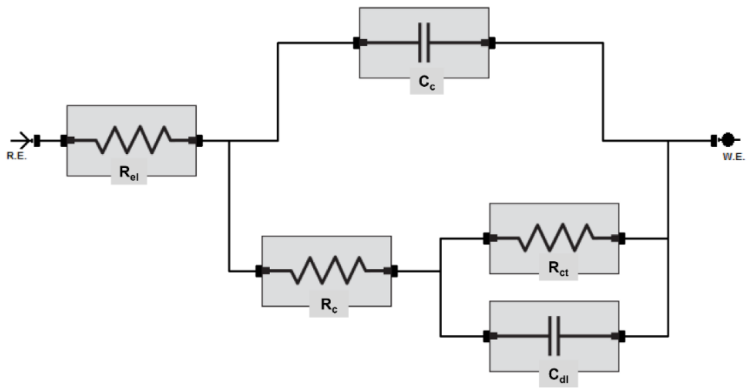


**Figure S5.** Equivalent circuit model used for fitting the EIS spectra.

**Table S1.** Circuit parameters calculated from the EIS spectra.

| | $R_{el}$ (Ohm) | $C_c$ ($10^{-5}$ F) | $R_c$ (Ohm) | $C_{dl}$ ($10^{-5}$ F) | $R_{ct}$ (kOhm) |
|---|---|---|---|---|---|
| **NA** | 1.62 | 8.19 | 263 | 3.70 | 14.5 |
| **NT10-S** | 1.10 | 4.24 | 650 | 3.10 | 23.3 |
| **NT40-S** | 1.70 | 5.45 | 120 | 7.22 | 4.43 |

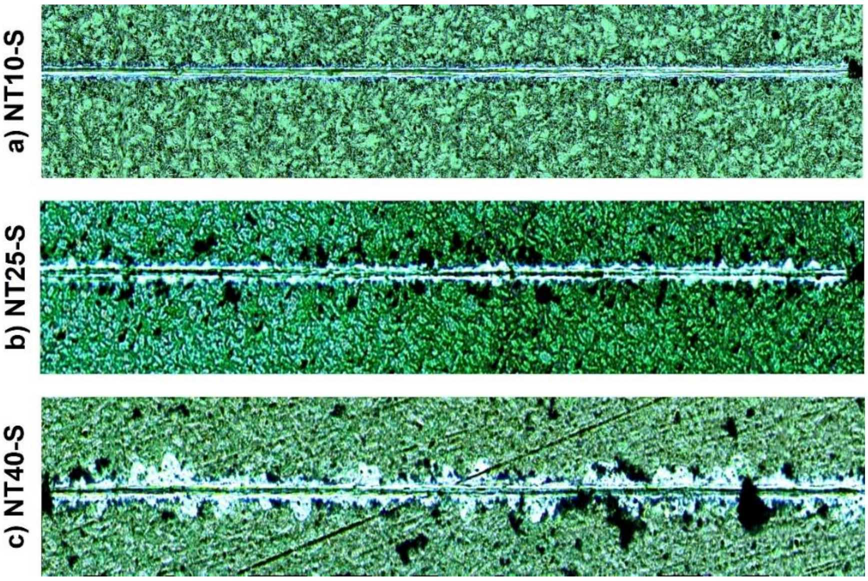


**Figure S6.** Images of the microscratched surfaces for a) NT10-S, b) NT25-S and c) NT40-S samples.
